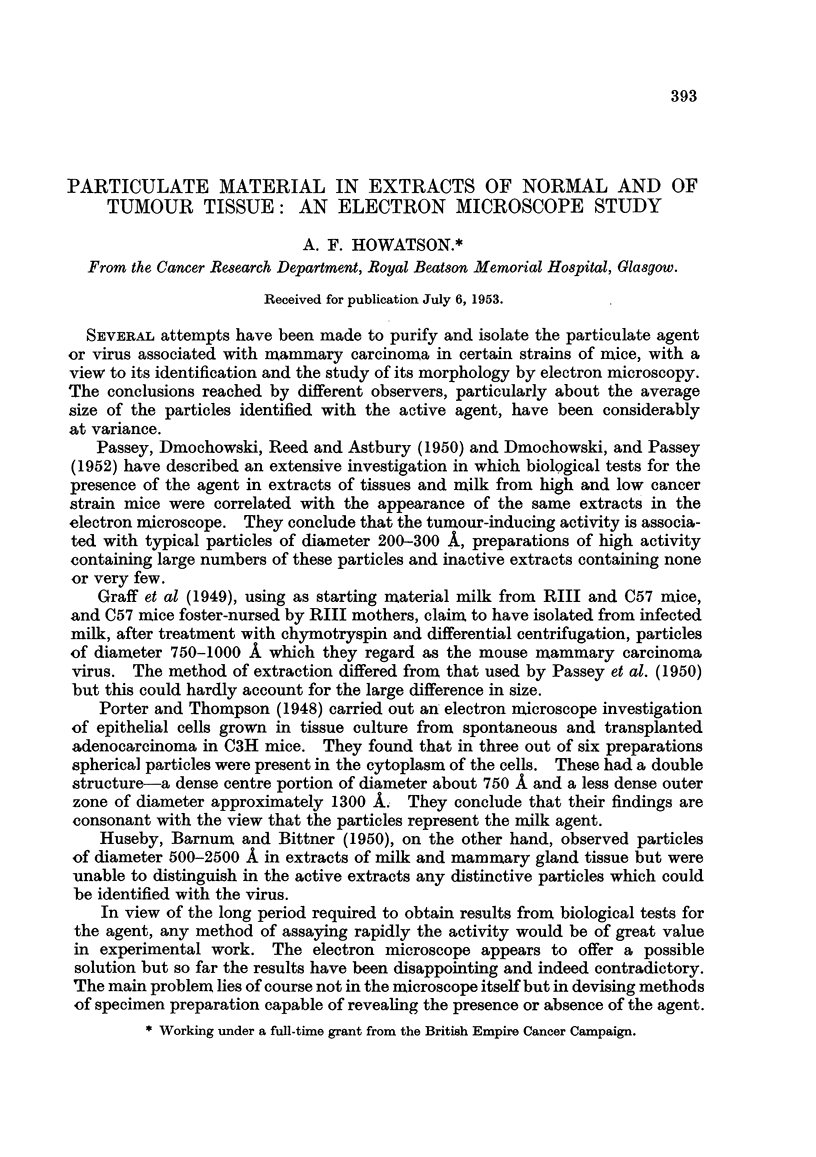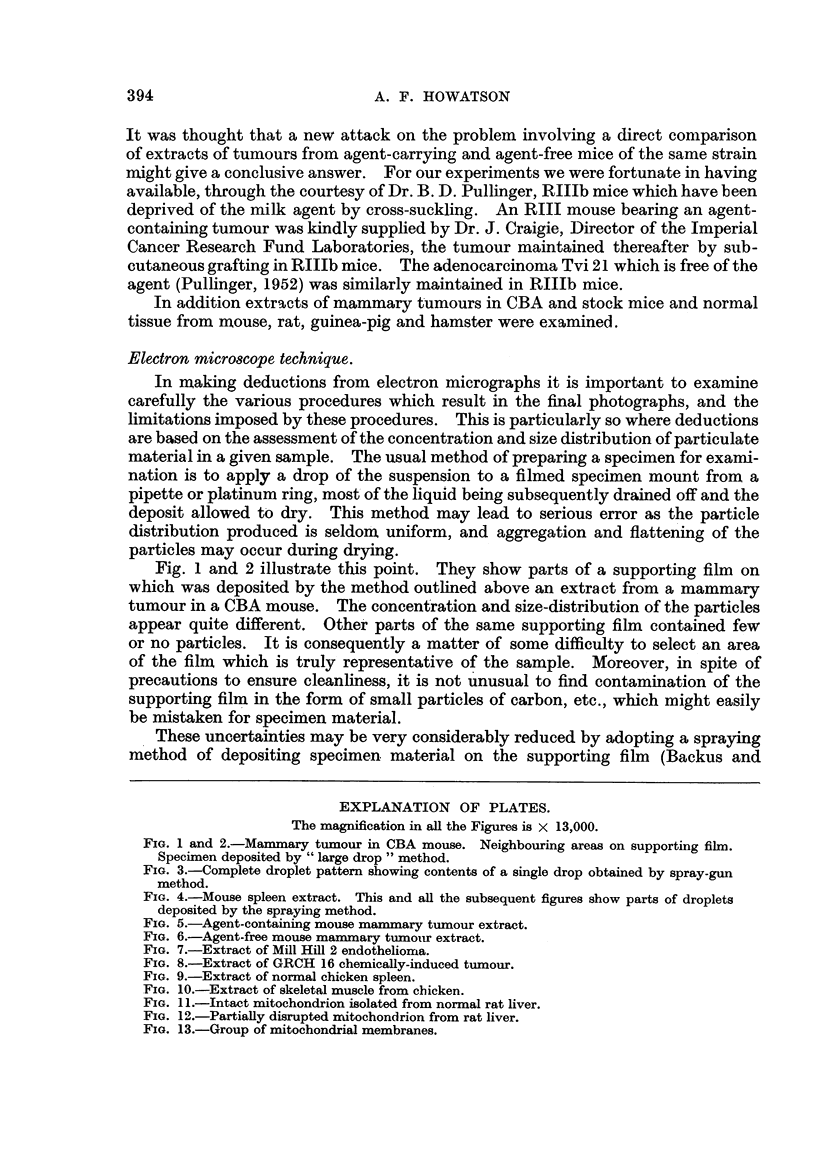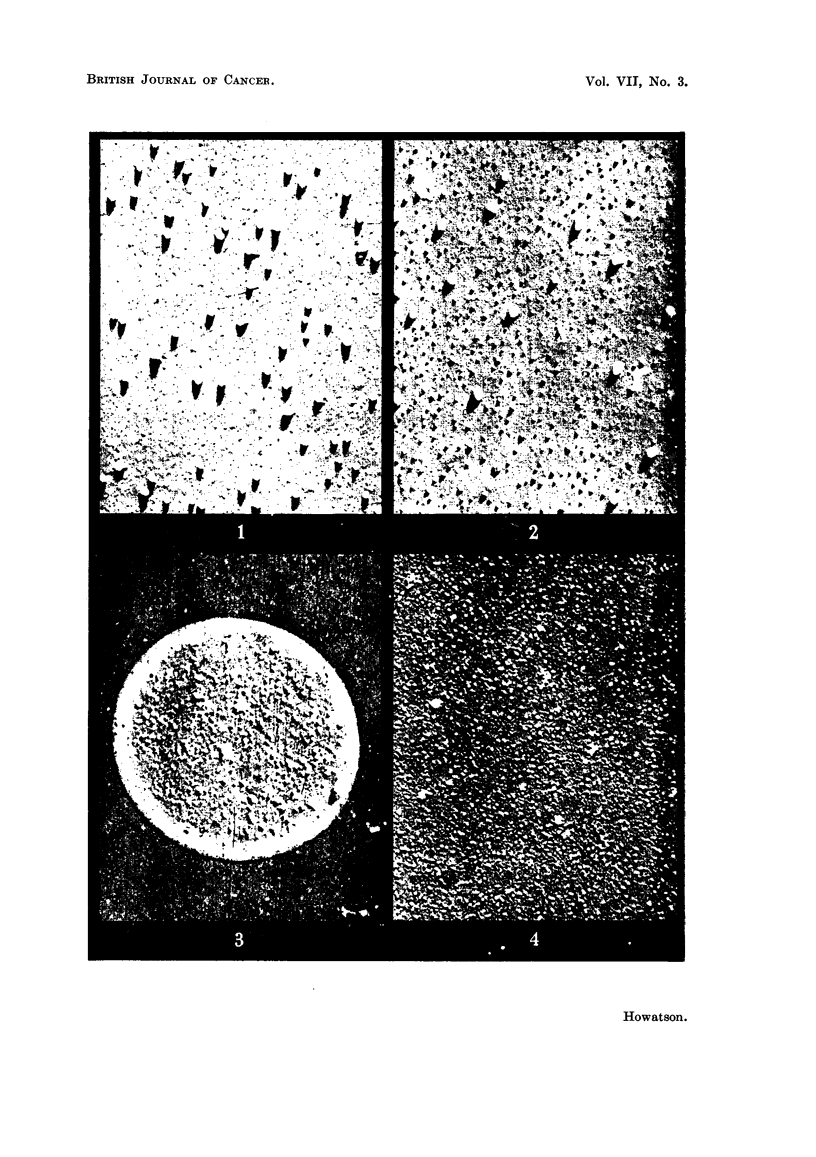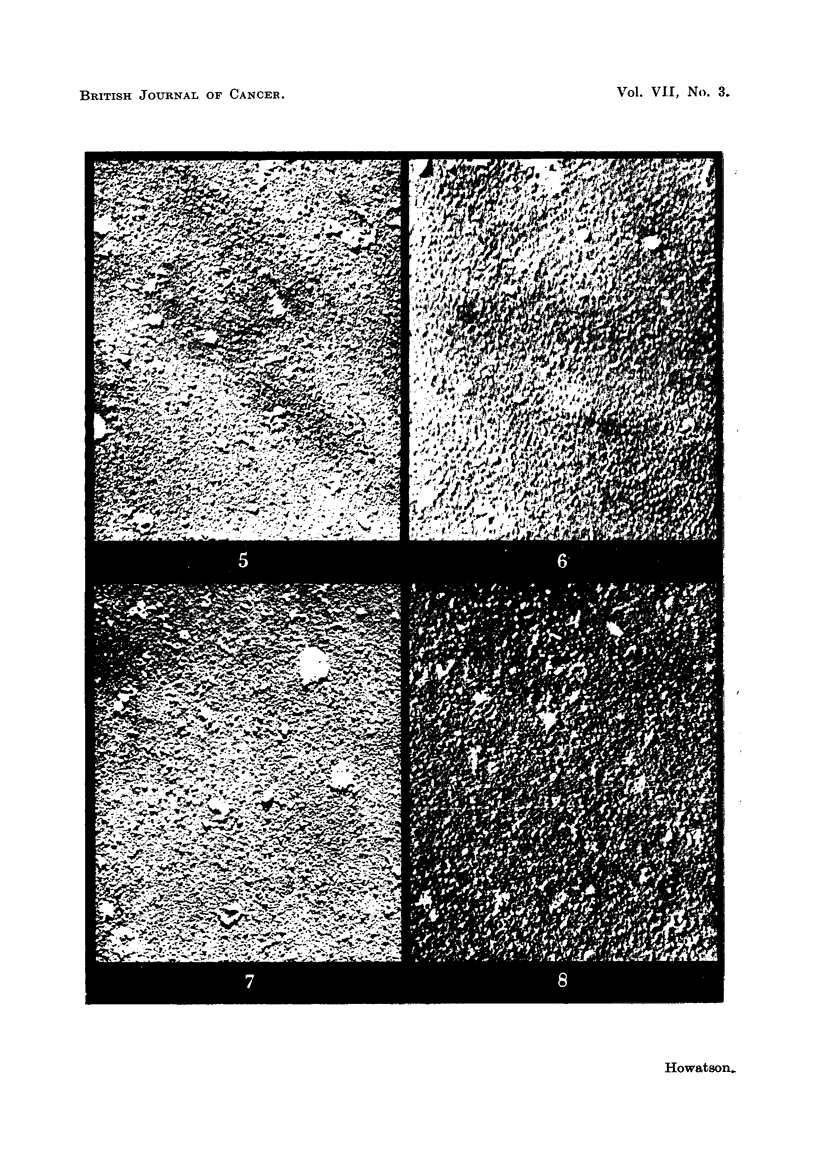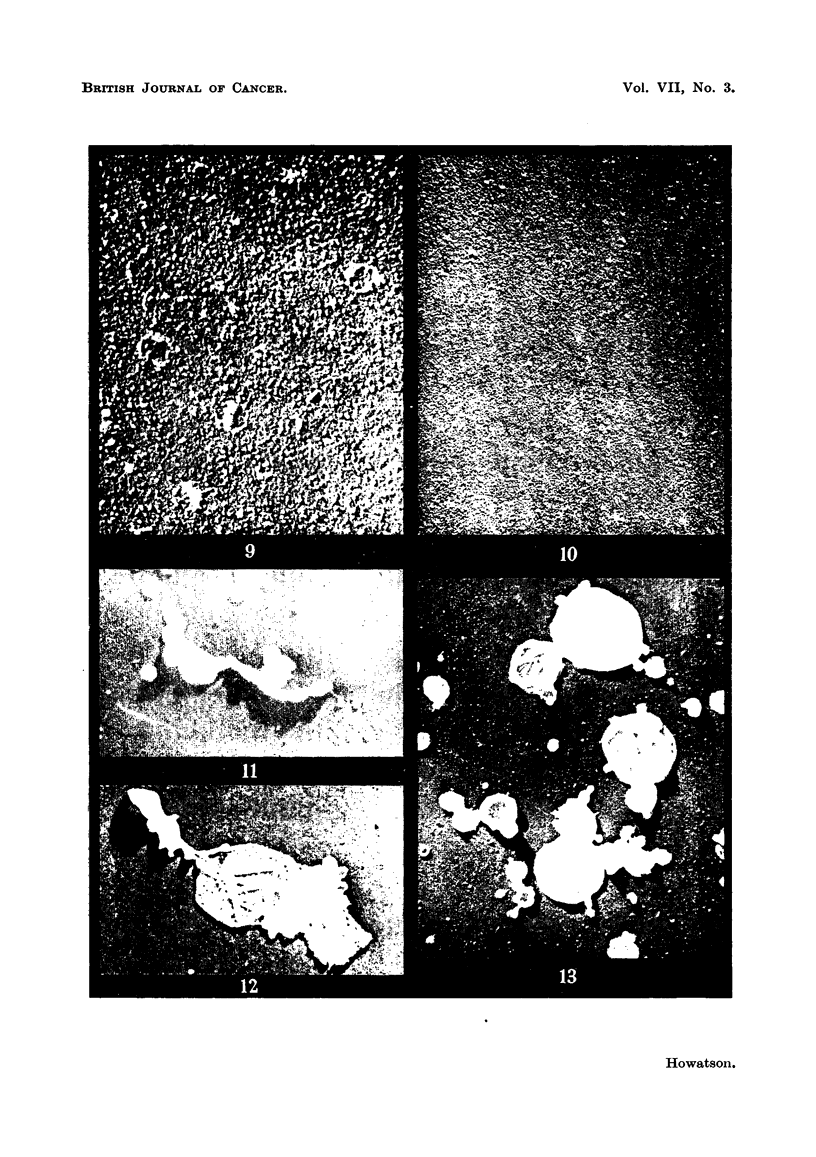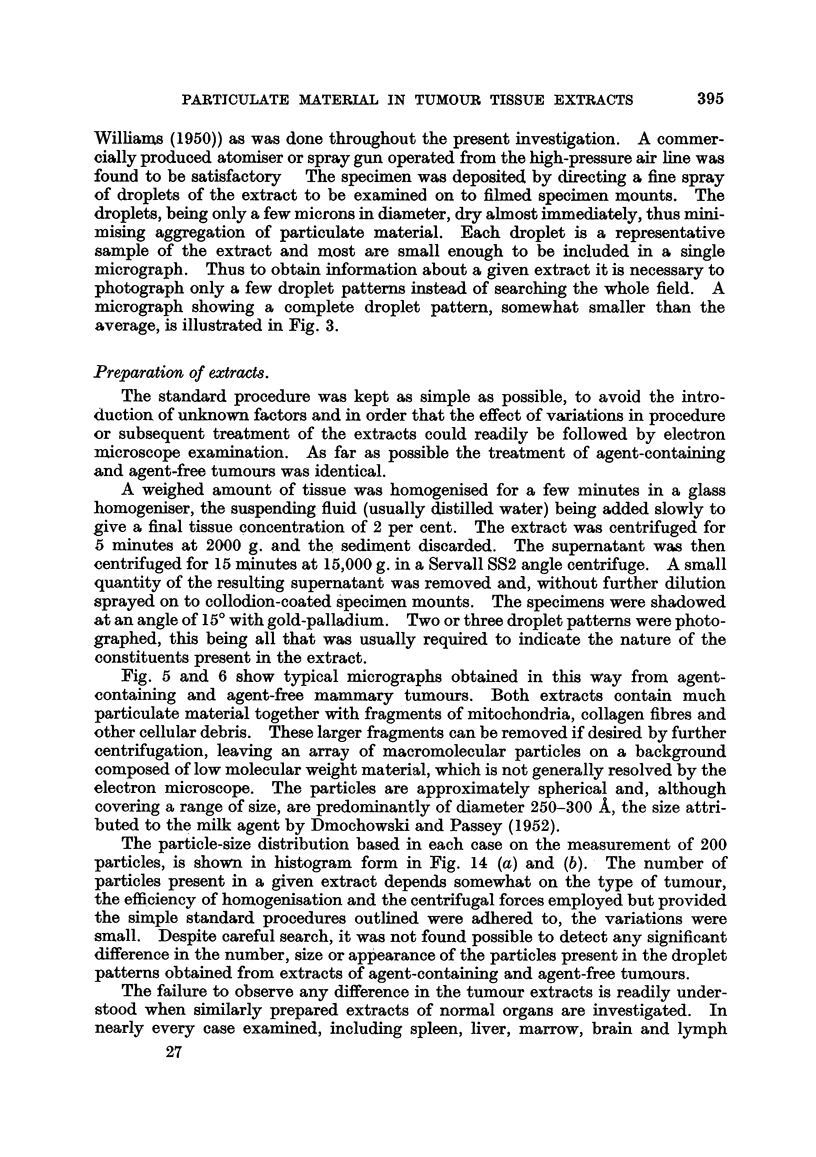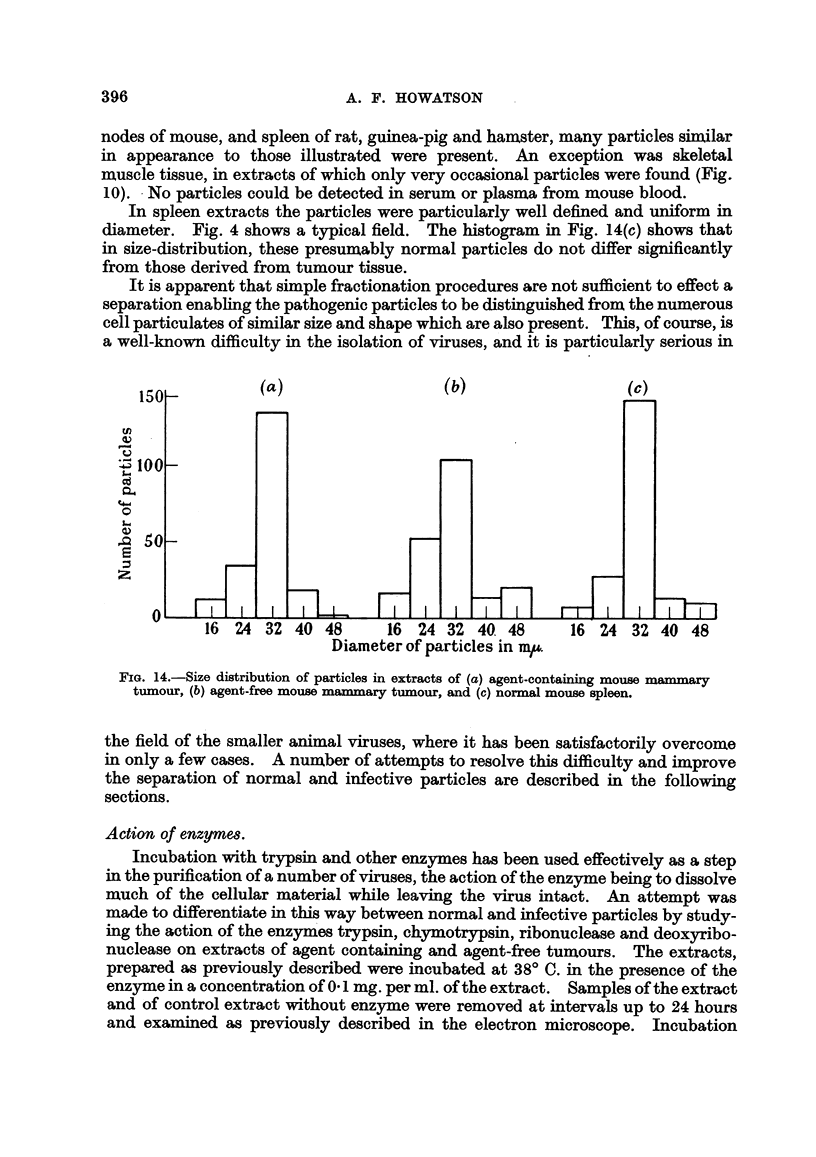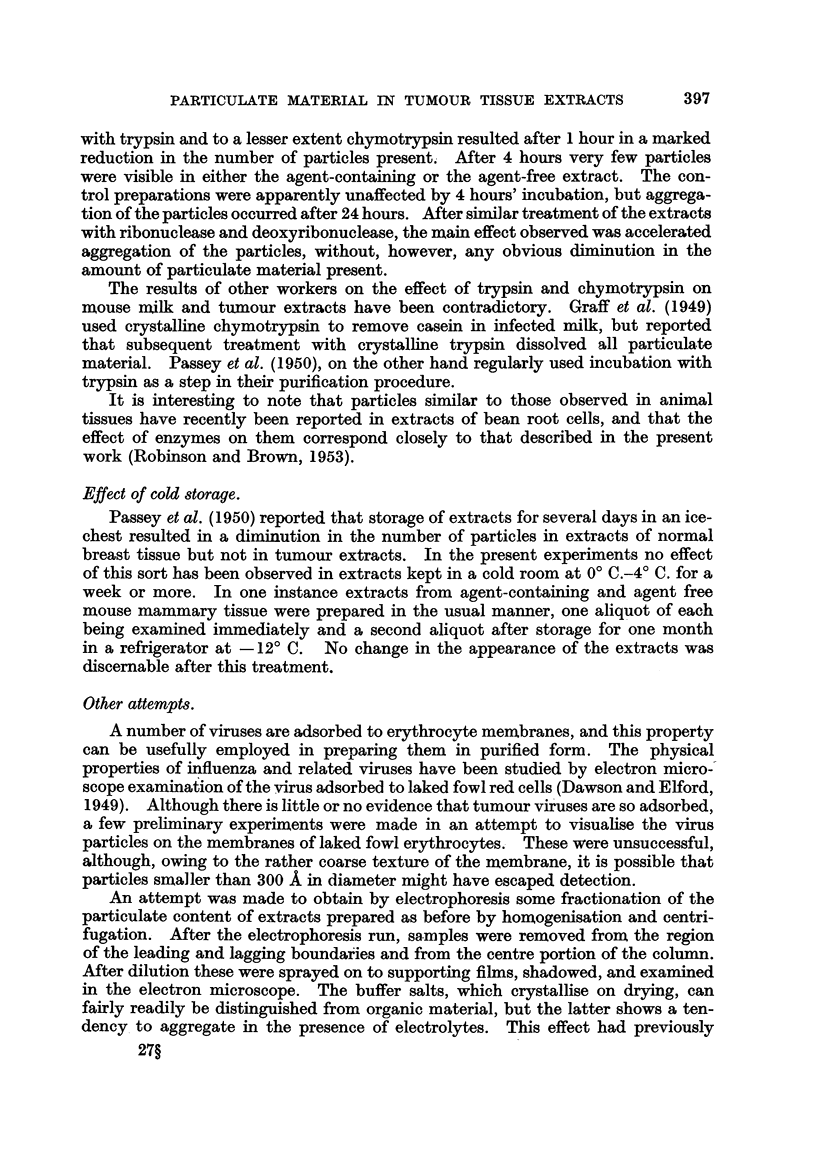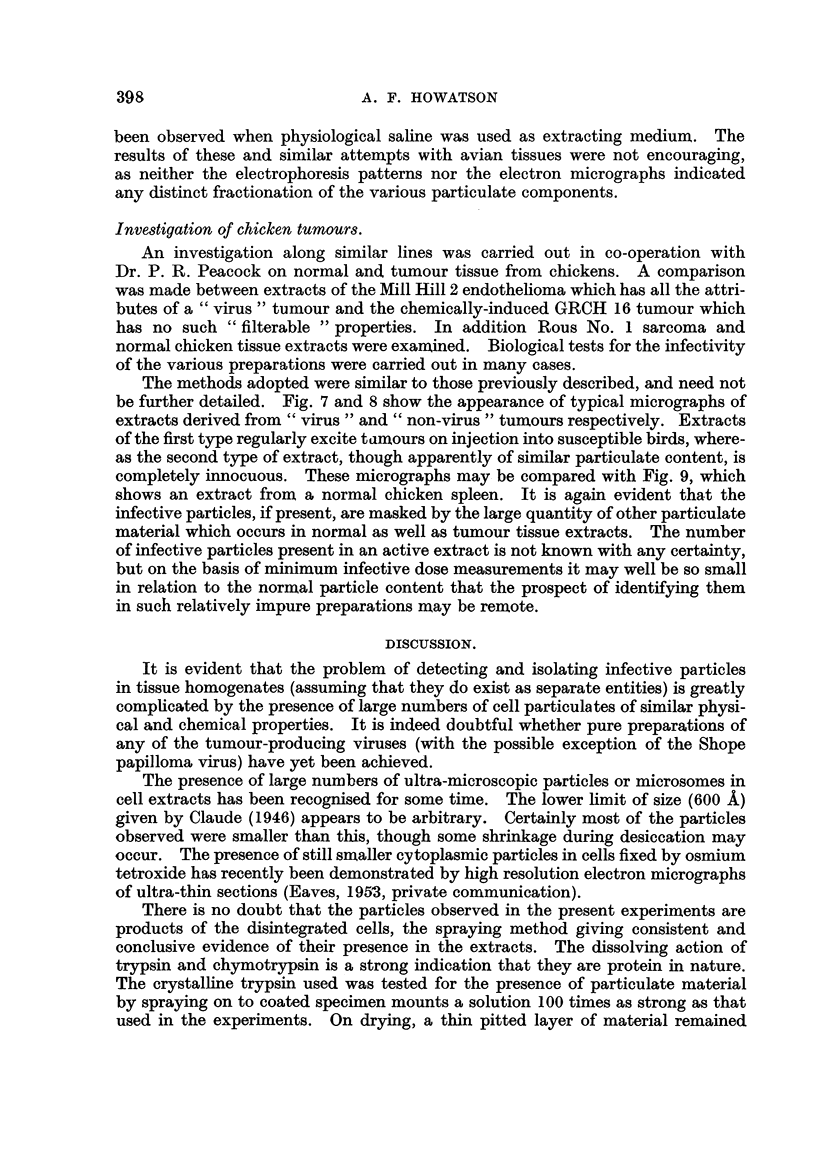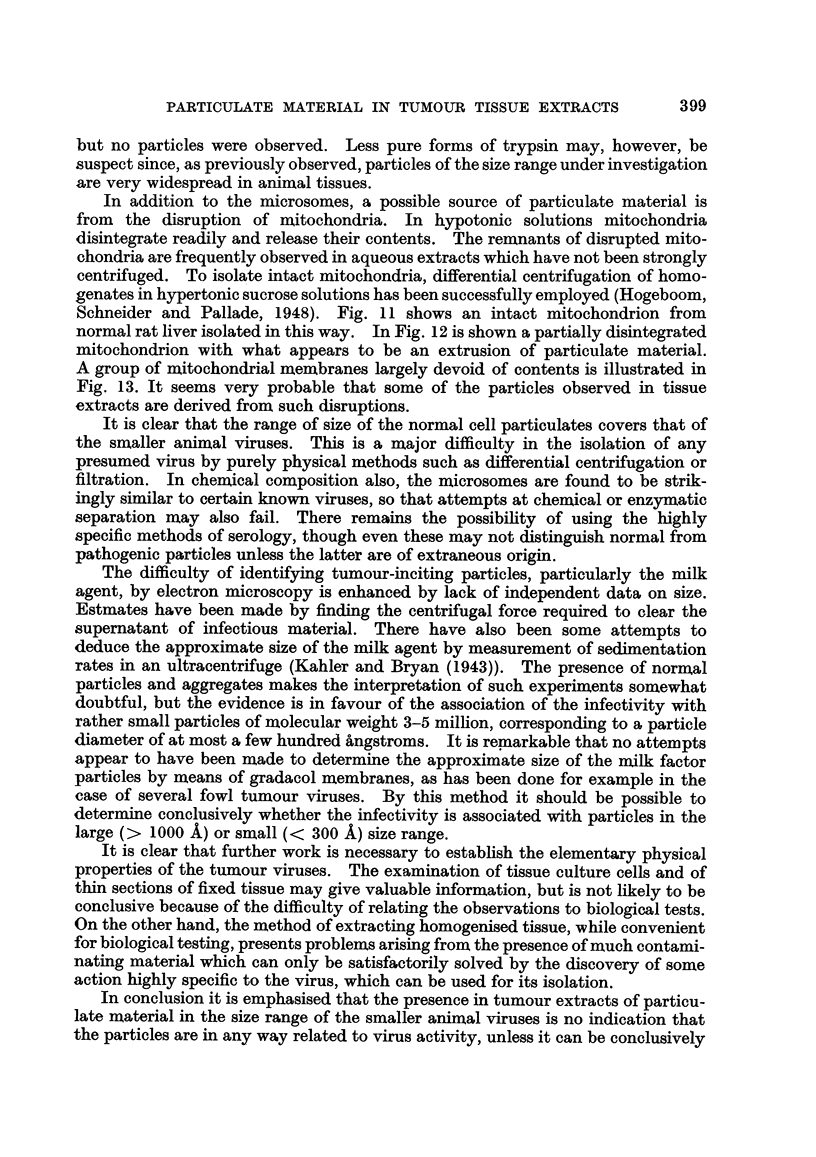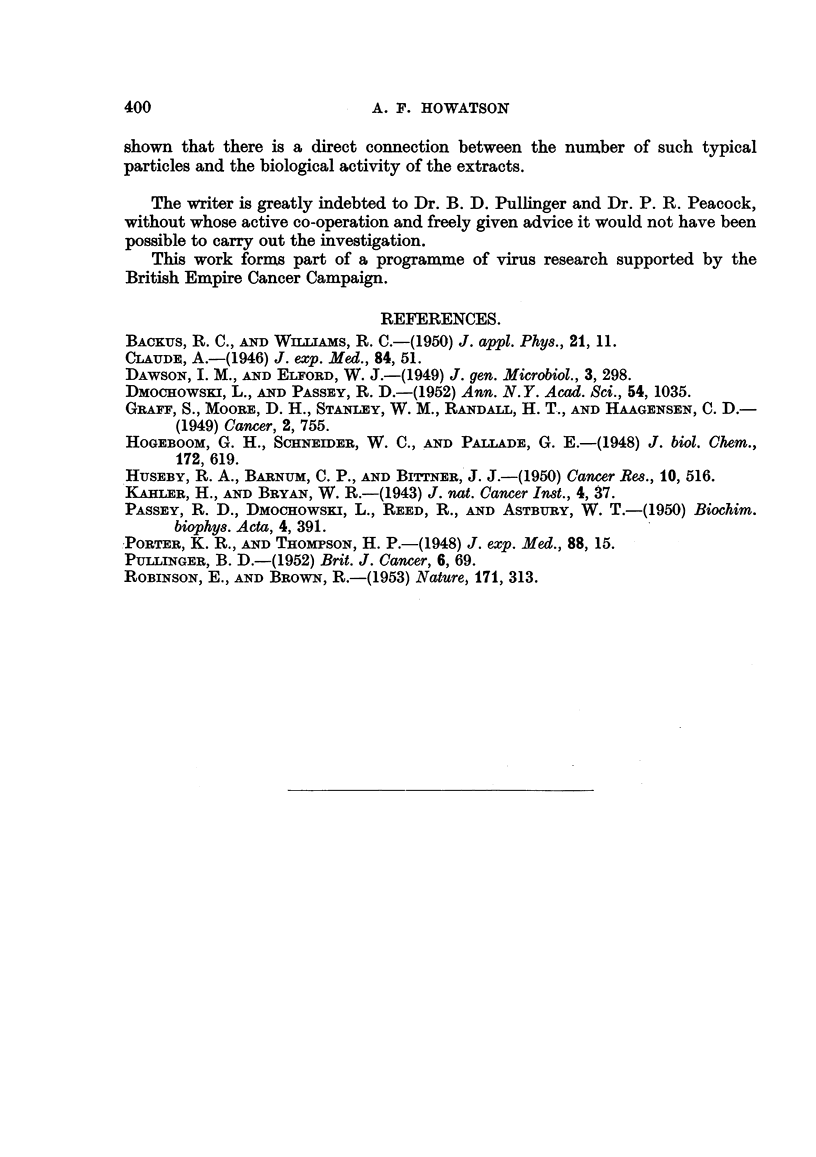# Particulate Material in Extracts of Normal and of Tumour Tissue: an Electron Microscope Study

**DOI:** 10.1038/bjc.1953.37

**Published:** 1953-09

**Authors:** A. F. Howatson

## Abstract

**Images:**


### PARTICULATE MATERIAL IN EXTRACTS OF NORMAL AND OF TUMOUR TISSUE: AN ELECTRON MICROSCOPE STUDY

A. F. HOWATSON.*

*From the Cancer Research Department, Royal Beatson Memorial Hospital, Glasgow.*

Received for publication July 6, 1953.

SEVERAL attempts have been made to purify and isolate the particulate agent or virus associated with mammary carcinoma in certain strains of mice, with a view to its identification and the study of its morphology by electron microscopy. The conclusions reached by different observers, particularly about the average size of the particles identified with the active agent, have been considerably at variance.

Passey, Dmochowski, Reed and Astbury (1950) and Dmochowski, and Passey (1952) have described an extensive investigation in which biological tests for the presence of the agent in extracts of tissues and milk from high and low cancer strain mice were correlated with the appearance of the same extracts in the electron microscope. They conclude that the tumour-inducing activity is associated with typical particles of diameter 200–300 Å, preparations of high activity containing large numbers of these particles and inactive extracts containing none or very few.

Graff *et al* (1949), using as starting material milk from RIII and C57 mice, and C57 mice foster-nursed by RIII mothers, claim to have isolated from infected milk, after treatment with chymotryspin and differential centrifugation, particles of diameter 750–1000 Å which they regard as the mouse mammary carcinoma virus. The method of extraction differed from that used by Passey *et al.* (1950) but this could hardly account for the large difference in size.

Porter and Thompson (1948) carried out an electron microscope investigation of epithelial cells grown in tissue culture from spontaneous and transplanted adenocarcinoma in C3H mice. They found that in three out of six preparations spherical particles were present in the cytoplasm of the cells. These had a double structure—a dense centre portion of diameter about 750 Å and a less dense outer zone of diameter approximately 1300 Å. They conclude that their findings are consonant with the view that the particles represent the milk agent.

Huseby, Barnum and Bittner (1950), on the other hand, observed particles of diameter 500–2500 Å in extracts of milk and mammary gland tissue but were unable to distinguish in the active extracts any distinctive particles which could be identified with the virus.

In view of the long period required to obtain results from biological tests for the agent, any method of assaying rapidly the activity would be of great value in experimental work. The electron microscope appears to offer a possible solution but so far the results have been disappointing and indeed contradictory. The main problem lies of course not in the microscope itself but in devising methods of specimen preparation capable of revealing the presence or absence of the agent.

* Working under a full-time grant from the British Empire Cancer Campaign.

It was thought that a new attack on the problem involving a direct comparison of extracts of tumours from agent-carrying and agent-free mice of the same strain might give a conclusive answer. For our experiments we were fortunate in having available, through the courtesy of Dr. B. D. Pullinger, RIIIb mice which have been deprived of the milk agent by cross-suckling. An RIII mouse bearing an agent-containing tumour was kindly supplied by Dr. J. Craigie, Director of the Imperial Cancer Research Fund Laboratories, the tumour maintained thereafter by subcutaneous grafting in RIIIb mice. The adenocarcinoma Tvi 21 which is free of the agent (Pullinger, 1952) was similarly maintained in RIIIb mice.

In addition extracts of mammary tumours in CBA and stock mice and normal tissue from mouse, rat, guinea-pig and hamster were examined.

*Electron microscope technique.*

In making deductions from electron micrographs it is important to examine carefully the various procedures which result in the final photographs, and the limitations imposed by these procedures. This is particularly so where deductions are based on the assessment of the concentration and size distribution of particulate material in a given sample. The usual method of preparing a specimen for examination is to apply a drop of the suspension to a filmed specimen mount from a pipette or platinum ring, most of the liquid being subsequently drained off and the deposit allowed to dry. This method may lead to serious error as the particle distribution produced is seldom uniform, and aggregation and flattening of the particles may occur during drying.

Fig. 1 and 2 illustrate this point. They show parts of a supporting film on which was deposited by the method outlined above an extract from a mammary tumour in a CBA mouse. The concentration and size-distribution of the particles appear quite different. Other parts of the same supporting film contained few or no particles. It is consequently a matter of some difficulty to select an area of the film which is truly representative of the sample. Moreover, in spite of precautions to ensure cleanliness, it is not unusual to find contamination of the supporting film in the form of small particles of carbon, etc., which might easily be mistaken for specimen material.

These uncertainties may be very considerably reduced by adopting a spraying method of depositing specimen material on the supporting film (Backus and

---

EXPLANATION OF PLATES.

The magnification in all the Figures is × 13,000.

FIG. 1 and 2.—Mammary tumour in CBA mouse. Neighbouring areas on supporting film. Specimen deposited by " large drop " method.

FIG. 3.—Complete droplet pattern showing contents of a single drop obtained by spray-gun method.

FIG. 4.—Mouse spleen extract. This and all the subsequent figures show parts of droplets deposited by the spraying method.

FIG. 5.—Agent-containing mouse mammary tumour extract.

FIG. 6.—Agent-free mouse mammary tumour extract.

FIG. 7.—Extract of Mill Hill 2 endothelioma.

FIG. 8.—Extract of GRCH 16 chemically-induced tumour.

FIG. 9.—Extract of normal chicken spleen.

FIG. 10.—Extract of skeletal muscle from chicken.

FIG. 11.—Intact mitochondrion isolated from normal rat liver.

FIG. 12.—Partially disrupted mitochondrion from rat liver.

FIG. 13.—Group of mitochondrial membranes.

Howatson.

Howatson.

Howatson.

Williams (1950)) as was done throughout the present investigation. A commercially produced atomiser or spray gun operated from the high-pressure air line was found to be satisfactory The specimen was deposited by directing a fine spray of droplets of the extract to be examined on to filmed specimen mounts. The droplets, being only a few microns in diameter, dry almost immediately, thus minimising aggregation of particulate material. Each droplet is a representative sample of the extract and most are small enough to be included in a single micrograph. Thus to obtain information about a given extract it is necessary to photograph only a few droplet patterns instead of searching the whole field. A micrograph showing a complete droplet pattern, somewhat smaller than the average, is illustrated in Fig. 3.

*Preparation of extracts.*

The standard procedure was kept as simple as possible, to avoid the introduction of unknown factors and in order that the effect of variations in procedure or subsequent treatment of the extracts could readily be followed by electron microscope examination. As far as possible the treatment of agent-containing and agent-free tumours was identical.

A weighed amount of tissue was homogenised for a few minutes in a glass homogeniser, the suspending fluid (usually distilled water) being added slowly to give a final tissue concentration of 2 per cent. The extract was centrifuged for 5 minutes at 2000 g. and the sediment discarded. The supernatant was then centrifuged for 15 minutes at 15,000 g. in a Servall SS2 angle centrifuge. A small quantity of the resulting supernatant was removed and, without further dilution sprayed on to collodion-coated specimen mounts. The specimens were shadowed at an angle of 15° with gold-palladium. Two or three droplet patterns were photographed, this being all that was usually required to indicate the nature of the constituents present in the extract.

Fig. 5 and 6 show typical micrographs obtained in this way from agent-containing and agent-free mammary tumours. Both extracts contain much particulate material together with fragments of mitochondria, collagen fibres and other cellular debris. These larger fragments can be removed if desired by further centrifugation, leaving an array of macromolecular particles on a background composed of low molecular weight material, which is not generally resolved by the electron microscope. The particles are approximately spherical and, although covering a range of size, are predominantly of diameter 250–300 Å, the size attributed to the milk agent by Dmochowski and Passey (1952).

The particle-size distribution based in each case on the measurement of 200 particles, is shown in histogram form in Fig. 14 (*a*) and (*b*). The number of particles present in a given extract depends somewhat on the type of tumour, the efficiency of homogenisation and the centrifugal forces employed but provided the simple standard procedures outlined were adhered to, the variations were small. Despite careful search, it was not found possible to detect any significant difference in the number, size or appearance of the particles present in the droplet patterns obtained from extracts of agent-containing and agent-free tumours.

The failure to observe any difference in the tumour extracts is readily understood when similarly prepared extracts of normal organs are investigated. In nearly every case examined, including spleen, liver, marrow, brain and lymph nodes of mouse, and spleen of rat, guinea-pig and hamster, many particles similar in appearance to those illustrated were present. An exception was skeletal muscle tissue, in extracts of which only very occasional particles were found (Fig. 10). No particles could be detected in serum or plasma from mouse blood.

In spleen extracts the particles were particularly well defined and uniform in diameter. Fig. 4 shows a typical field. The histogram in Fig. 14(*c*) shows that in size-distribution, these presumably normal particles do not differ significantly from those derived from tumour tissue.

It is apparent that simple fractionation procedures are not sufficient to effect a separation enabling the pathogenic particles to be distinguished from the numerous cell particulates of similar size and shape which are also present. This, of course, is a well-known difficulty in the isolation of viruses, and it is particularly serious in

FIG. 14.—Size distribution of particles in extracts of (*a*) agent-containing mouse mammary tumour, (*b*) agent-free mouse mammary tumour, and (*c*) normal mouse spleen.

the field of the smaller animal viruses, where it has been satisfactorily overcome in only a few cases. A number of attempts to resolve this difficulty and improve the separation of normal and infective particles are described in the following sections.

*Action of enzymes.*

Incubation with trypsin and other enzymes has been used effectively as a step in the purification of a number of viruses, the action of the enzyme being to dissolve much of the cellular material while leaving the virus intact. An attempt was made to differentiate in this way between normal and infective particles by studying the action of the enzymes trypsin, chymotrypsin, ribonuclease and deoxyribonuclease on extracts of agent containing and agent-free tumours. The extracts, prepared as previously described were incubated at 38° C. in the presence of the enzyme in a concentration of 0·1 mg. per ml. of the extract. Samples of the extract and of control extract without enzyme were removed at intervals up to 24 hours and examined as previously described in the electron microscope. Incubation with trypsin and to a lesser extent chymotrypsin resulted after 1 hour in a marked reduction in the number of particles present. After 4 hours very few particles were visible in either the agent-containing or the agent-free extract. The control preparations were apparently unaffected by 4 hours' incubation, but aggregation of the particles occurred after 24 hours. After similar treatment of the extracts with ribonuclease and deoxyribonuclease, the main effect observed was accelerated aggregation of the particles, without, however, any obvious diminution in the amount of particulate material present.

The results of other workers on the effect of trypsin and chymotrypsin on mouse milk and tumour extracts have been contradictory. Graff *et al.* (1949) used crystalline chymotrypsin to remove casein in infected milk, but reported that subsequent treatment with crystalline trypsin dissolved all particulate material. Passey *et al.* (1950), on the other hand regularly used incubation with trypsin as a step in their purification procedure.

It is interesting to note that particles similar to those observed in animal tissues have recently been reported in extracts of bean root cells, and that the effect of enzymes on them correspond closely to that described in the present work (Robinson and Brown, 1953).

*Effect of cold storage.*

Passey *et al.* (1950) reported that storage of extracts for several days in an ice-chest resulted in a diminution in the number of particles in extracts of normal breast tissue but not in tumour extracts. In the present experiments no effect of this sort has been observed in extracts kept in a cold room at 0° C.–4° C. for a week or more. In one instance extracts from agent-containing and agent free mouse mammary tissue were prepared in the usual manner, one aliquot of each being examined immediately and a second aliquot after storage for one month in a refrigerator at $-12°$ C. No change in the appearance of the extracts was discernable after this treatment.

*Other attempts.*

A number of viruses are adsorbed to erythrocyte membranes, and this property can be usefully employed in preparing them in purified form. The physical properties of influenza and related viruses have been studied by electron microscope examination of the virus adsorbed to laked fowl red cells (Dawson and Elford, 1949). Although there is little or no evidence that tumour viruses are so adsorbed, a few preliminary experiments were made in an attempt to visualise the virus particles on the membranes of laked fowl erythrocytes. These were unsuccessful, although, owing to the rather coarse texture of the membrane, it is possible that particles smaller than 300 Å in diameter might have escaped detection.

An attempt was made to obtain by electrophoresis some fractionation of the particulate content of extracts prepared as before by homogenisation and centrifugation. After the electrophoresis run, samples were removed from the region of the leading and lagging boundaries and from the centre portion of the column. After dilution these were sprayed on to supporting films, shadowed, and examined in the electron microscope. The buffer salts, which crystallise on drying, can fairly readily be distinguished from organic material, but the latter shows a tendency to aggregate in the presence of electrolytes. This effect had previously been observed when physiological saline was used as extracting medium. The results of these and similar attempts with avian tissues were not encouraging, as neither the electrophoresis patterns nor the electron micrographs indicated any distinct fractionation of the various particulate components.

*Investigation of chicken tumours.*

An investigation along similar lines was carried out in co-operation with Dr. P. R. Peacock on normal and tumour tissue from chickens. A comparison was made between extracts of the Mill Hill 2 endothelioma which has all the attributes of a " virus " tumour and the chemically-induced GRCH 16 tumour which has no such " filterable " properties. In addition Rous No. 1 sarcoma and normal chicken tissue extracts were examined. Biological tests for the infectivity of the various preparations were carried out in many cases.

The methods adopted were similar to those previously described, and need not be further detailed. Fig. 7 and 8 show the appearance of typical micrographs of extracts derived from " virus " and " non-virus " tumours respectively. Extracts of the first type regularly excite tumours on injection into susceptible birds, whereas the second type of extract, though apparently of similar particulate content, is completely innocuous. These micrographs may be compared with Fig. 9, which shows an extract from a normal chicken spleen. It is again evident that the infective particles, if present, are masked by the large quantity of other particulate material which occurs in normal as well as tumour tissue extracts. The number of infective particles present in an active extract is not known with any certainty, but on the basis of minimum infective dose measurements it may well be so small in relation to the normal particle content that the prospect of identifying them in such relatively impure preparations may be remote.

### DISCUSSION.

It is evident that the problem of detecting and isolating infective particles in tissue homogenates (assuming that they do exist as separate entities) is greatly complicated by the presence of large numbers of cell particulates of similar physical and chemical properties. It is indeed doubtful whether pure preparations of any of the tumour-producing viruses (with the possible exception of the Shope papilloma virus) have yet been achieved.

The presence of large numbers of ultra-microscopic particles or microsomes in cell extracts has been recognised for some time. The lower limit of size (600 Å) given by Claude (1946) appears to be arbitrary. Certainly most of the particles observed were smaller than this, though some shrinkage during desiccation may occur. The presence of still smaller cytoplasmic particles in cells fixed by osmium tetroxide has recently been demonstrated by high resolution electron micrographs of ultra-thin sections (Eaves, 1953, private communication).

There is no doubt that the particles observed in the present experiments are products of the disintegrated cells, the spraying method giving consistent and conclusive evidence of their presence in the extracts. The dissolving action of trypsin and chymotrypsin is a strong indication that they are protein in nature. The crystalline trypsin used was tested for the presence of particulate material by spraying on to coated specimen mounts a solution 100 times as strong as that used in the experiments. On drying, a thin pitted layer of material remained but no particles were observed. Less pure forms of trypsin may, however, be suspect since, as previously observed, particles of the size range under investigation are very widespread in animal tissues.

In addition to the microsomes, a possible source of particulate material is from the disruption of mitochondria. In hypotonic solutions mitochondria disintegrate readily and release their contents. The remnants of disrupted mitochondria are frequently observed in aqueous extracts which have not been strongly centrifuged. To isolate intact mitochondria, differential centrifugation of homogenates in hypertonic sucrose solutions has been successfully employed (Hogeboom, Schneider and Pallade, 1948). Fig. 11 shows an intact mitochondrion from normal rat liver isolated in this way. In Fig. 12 is shown a partially disintegrated mitochondrion with what appears to be an extrusion of particulate material. A group of mitochondrial membranes largely devoid of contents is illustrated in Fig. 13. It seems very probable that some of the particles observed in tissue extracts are derived from such disruptions.

It is clear that the range of size of the normal cell particulates covers that of the smaller animal viruses. This is a major difficulty in the isolation of any presumed virus by purely physical methods such as differential centrifugation or filtration. In chemical composition also, the microsomes are found to be strikingly similar to certain known viruses, so that attempts at chemical or enzymatic separation may also fail. There remains the possibility of using the highly specific methods of serology, though even these may not distinguish normal from pathogenic particles unless the latter are of extraneous origin.

The difficulty of identifying tumour-inciting particles, particularly the milk agent, by electron microscopy is enhanced by lack of independent data on size. Estmates have been made by finding the centrifugal force required to clear the supernatant of infectious material. There have also been some attempts to deduce the approximate size of the milk agent by measurement of sedimentation rates in an ultracentrifuge (Kahler and Bryan (1943)). The presence of normal particles and aggregates makes the interpretation of such experiments somewhat doubtful, but the evidence is in favour of the association of the infectivity with rather small particles of molecular weight 3–5 million, corresponding to a particle diameter of at most a few hundred ångstroms. It is remarkable that no attempts appear to have been made to determine the approximate size of the milk factor particles by means of gradacol membranes, as has been done for example in the case of several fowl tumour viruses. By this method it should be possible to determine conclusively whether the infectivity is associated with particles in the large ($>$ 1000 Å) or small ($<$ 300 Å) size range.

It is clear that further work is necessary to establish the elementary physical properties of the tumour viruses. The examination of tissue culture cells and of thin sections of fixed tissue may give valuable information, but is not likely to be conclusive because of the difficulty of relating the observations to biological tests. On the other hand, the method of extracting homogenised tissue, while convenient for biological testing, presents problems arising from the presence of much contaminating material which can only be satisfactorily solved by the discovery of some action highly specific to the virus, which can be used for its isolation.

In conclusion it is emphasised that the presence in tumour extracts of particulate material in the size range of the smaller animal viruses is no indication that the particles are in any way related to virus activity, unless it can be conclusively shown that there is a direct connection between the number of such typical particles and the biological activity of the extracts.

The writer is greatly indebted to Dr. B. D. Pullinger and Dr. P. R. Peacock, without whose active co-operation and freely given advice it would not have been possible to carry out the investigation.

This work forms part of a programme of virus research supported by the British Empire Cancer Campaign.

### REFERENCES.

Backus, R. C., and Williams, R. C.—(1950) *J. appl. Phys.*, **21**, 11.
Claude, A.—(1946) *J. exp. Med.*, **84**, 51.
Dawson, I. M., and Elford, W. J.—(1949) *J. gen. Microbiol.*, **3**, 298.
Dmochowski, L., and Passey, R. D.—(1952) *Ann. N.Y. Acad. Sci.*, **54**, 1035.
Graff, S., Moore, D. H., Stanley, W. M., Randall, H. T., and Haagensen, C. D.—(1949) *Cancer*, **2**, 755.
Hogeboom, G. H., Schneider, W. C., and Pallade, G. E.—(1948) *J. biol. Chem.*, **172**, 619.
Huseby, R. A., Barnum, C. P., and Bittner, J. J.—(1950) *Cancer Res.*, **10**, 516.
Kahler, H., and Bryan, W. R.—(1943) *J. nat. Cancer Inst.*, **4**, 37.
Passey, R. D., Dmochowski, L., Reed, R., and Astbury, W. T.—(1950) *Biochim. biophys. Acta*, **4**, 391.
Porter, K. R., and Thompson, H. P.—(1948) *J. exp. Med.*, **88**, 15.
Pullinger, B. D.—(1952) *Brit. J. Cancer*, **6**, 69.
Robinson, E., and Brown, R.—(1953) *Nature*, **171**, 313.